# HIV Silencing and Inducibility Are Heterogeneous and Are Affected by Factors Intrinsic to the Virus

**DOI:** 10.1128/mBio.00188-19

**Published:** 2019-06-25

**Authors:** Nicholas J. Norton, Hoi Ping Mok, Fatima Sharif, Jack C. Hirst, Andrew M. L. Lever

**Affiliations:** a Department of Medicine, University of Cambridge, Cambridge, United Kingdom; b Yong Loo Lin School of Medicine, National University of Singapore, Singapore; Albert Einstein College of Medicine; Albert Einstein College of Medicine

**Keywords:** HIV, inducibility, latency

## Abstract

Transcriptionally silent HIV proviruses form the major obstacle to eradicating HIV. Many studies of HIV latency have focused on the cellular mechanisms that maintain silencing of proviral DNA. Here we show that viral sequence variation affecting replicative ability leads to variable rates of silencing and ability to reactivate. We studied naturally occurring and engineered polymorphisms in a recently identified exonic splice enhancer (ESE_tat_) that regulates *tat* mRNA splicing and constructed viruses with increased (strain M1), reduced (strain M2), or completely absent (strain ERK) binding of splicing factors essential for optimal production of *tat* mRNA resulting in a corresponding change in Tat activity. The mutations affected viral replication, with M1 having wild-type (WT) kinetics, M2 exhibiting reduced kinetics, and ERK showing completely abrogated replication. Using single-round infection with green fluorescent protein (GFP)-expressing viruses to study proviral gene expression, we observed progressively greater rates of silencing relating to the degree of ESE_tat_ disruption, with the WT strain at 53%, strain M2 at 69%, and strain ERK at 94%. By stimulating infected cells with a latency reversal agent (phorbol myristate acetate [PMA], panobinostat, or JQ1), we observed that the dose required to achieve 50% of the maximum signal was lowest in the WT, intermediate in M2, and highest in ERK, indicating progressively higher thresholds for reactivation. These results suggest that the ability of silent proviruses to reactivate from latency is variable and that minor differences in the viral sequence can alter the proportion of silenced viruses as well as the threshold required to induce silenced viruses to reactivate and express.

## Abstract (precis)

A reservoir of infected cells in which the HIV genome is transcriptionally silent is acknowledged to be the principal barrier to eradicating the virus from an infected person. A number of cellular processes are implicated in this silencing; however, the viral factors that may contribute remain underexplored. Here we examined mutations altering the correct splicing of HIV gene products as a model to study whether differences in viral sequence can affect either the proportion of viruses that are active or silent or their ability to reactivate. We found that some naturally occurring variations result in viruses that are silenced at a higher rate and require a proportionally increased stimulus for reactivation from latency. These data suggest that the silencing and reactivation behavior of HIV exists in a spectrum, influenced by factors intrinsic to the virus.

## INTRODUCTION

HIV establishes latency in a population of infected cells where the virus has integrated its genome but no viral gene expression occurs. If they are genetically intact, these integrated proviruses are capable of later reactivation, generating virions which, in the absence of antiretroviral therapy, rekindle the infection. The latent reservoir is therefore a barrier to HIV cure, and the factors affecting HIV silencing and reactivation are subjects of intense research interest.

The biological processes involved in the maintenance of HIV silencing are heterogeneous. Cellular responses that silence HIV proviral transcription by modification of the local histone environment or by altering the availability of critical transcription factors are well studied (1–3). In contrast, little is known about virus-dependent factors that affect silencing behavior. Similarly, whether the ability of a mixed population of silenced viruses to be induced is a binary phenotype (“inducible” or “noninducible”) or a nonbinary phenotype (“more inducible” or “less inducible”) has not previously been explored.

The HIV transactivator protein Tat is an early viral gene product required for efficient downstream viral gene expression. Nascent viral RNA at the viral promoter adopts a helix-loop structure, *trans*-activation response element (TAR), to which Tat binds and enhances processivity of RNA polymerase II (4). The Tat-TAR interaction thus forms a positive-feedback axis and is important for stable viral gene expression (5). The *tat* gene consists of two exons and is encoded by two or more multiply spliced viral mRNAs. There are a number of splice variants, the predominant *tat* mRNA being Tat1 (6). Recently, an exonic splice enhancer (ESE_tat_) responsible for balanced splicing of *tat* mRNA was identified (7). Mutations profoundly disrupting ESE_tat_ abrogate splicing factor binding and alter *tat* mRNA splicing, causing a severe replication defect and very limited Tat protein production (7).

Natural variations in any of a number of mechanisms involved in HIV proviral transcription are predicted to alter silencing. Here we studied polymorphisms in the ESE_tat_ regions of full-length viral sequences to explore whether apparently intact HIV proviruses may exhibit different silencing behaviors due to altered Tat splicing. We found that the more extensive was the disruption of ESE_tat,_ the smaller the proportion of proviruses that expressed spontaneously, concomitant with a reduction in viral replication capacity. The concentration of latency reversal agents required to induce expression from the same proportion of silent proviruses also increases with increasing levels of disruption of ESE_tat_, indicating a higher threshold for induction. We thus provide an example where the ability of silent HIV to be induced is not a binary phenotype but represents a spectrum of inducibility determined by factors intrinsic to the virus.

## RESULTS

### ESE_tat_ is conserved in HIV-1.

**FIG 1:**
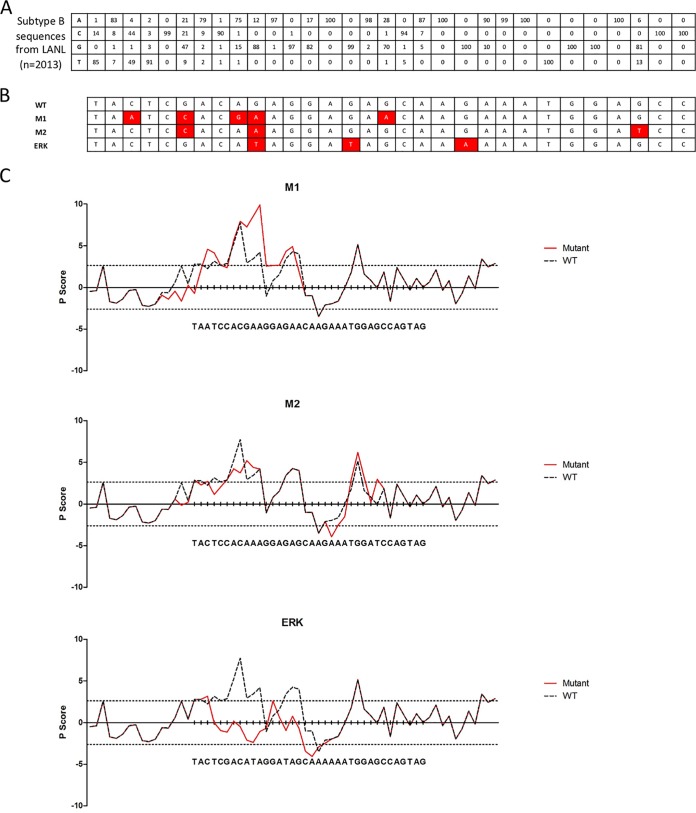
Predicted splicing factor binding of ESE_tat_ variants found *in vivo*. (A) ESE_tat_ is conserved among 2,013 subtype B sequences deposited in the Los Alamos National Laboratory HIV database. Subtype B sequences were aligned using HIVAlign (available from the database). The figure shows the percentage of identity for each base in the ESE_tat_ region. While variations exist, the majority of bases were 90% to 100% conserved. (B) Variant ESE_tat_ sequences of strains M1 and M2 and the mutant (ERK) created by Erkelenz et al. and used in this study. (C) Variant ESE_tat_ sequences have altered predicted splicing factor binding. Graphs show predicted splicing factor binding data calculated using the PESX score for each base; red lines show the scores for each mutant and dashed lines the scores for the wild type. The differences in P scores between the wild-type strain and the individual mutant strains were 13.9, −5.3, and −55.8 for the M1, M2, and ERK sequences, respectively.

To examine whether polymorphisms in the ESE_tat_ region occur *in vivo*, we examined full-length HIV subtype B sequences from the Los Alamos database (http://www.hiv.lanl.gov/). A total of 2,013 sequences were identified. Panel A of Fig. 1 shows the percentage of base identity for each position in ESE_tat_. The majority of positions showed greater than 90% conservation; however, some demonstrated a higher degree of polymorphism. This suggests that although evolutionary pressure constrains sequences in the ESE_tat_ region, there are viable sequence variants that can be found.

To study the effect of ESE_tat_ on HIV gene expression, we created mutants M1 and M2 (Fig. 1B). Circulating viruses bearing the ESE_tat_ sequences in M2 (8) and latent, noninducible viruses bearing the sequences in M1 have been described previously (9). We also created a mutant (termed ERK) which contains the same mutations as those demonstrated by Erkelenz et al. to maximally disrupt ESE_tat_ function (7). We used the putative exonic splicing enhancer/silencer (PESX) score (10) to predict splicing factor binding, using HIV_NL4–3_ as the reference wild-type (WT) viral sequence. The differences in P scores between the wild type and the mutants were 13.9, −5.3, and −55.8 for the M1, M2, and ERK sequences, respectively, where a positive score or a negative score corresponds to predicted increased or decreased splicing factor binding, respectively, compared to the wild type (Fig. 1C). Thus, M1 was predicted to exhibit a higher level of splicing factor binding and M2 a reduced level of splicing factor binding, with the predicted level of splicing factor binding lower still in ERK.

### ESE_tat_ disruption alters the relative proportions of *tat* mRNA species.

**FIG 2:**
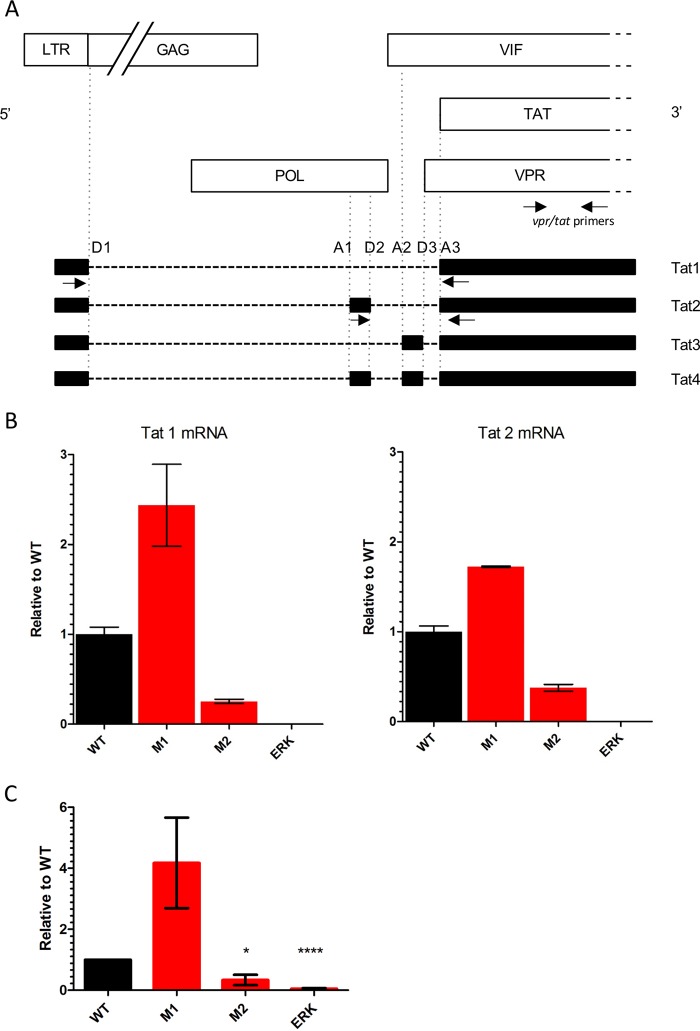
Effect of the mutations on *tat* mRNA splicing and Tat activity. (A) Schematic representation of the HIV genome showing patterns of alternative splicing generating different isoforms of *tat* mRNA. The inclusion of small exons gives rise to unique splice junctions. Arrows show the locations of primers used for qPCR to detect Tat1 and Tat 2. An all-*tat*/*vpr* primer set which detects all isoforms of *tat* and *vpr* was also used. (B) Variants in ESE_tat_ alter expression of *tat* mRNA. Jurkat cells were infected with WT and mutant viruses. The abundances of Tat1 and Tat2 were determined by qPCR and normalized to that of all *tat*/*vpr* transcripts. The graph shows the levels of Tat1 and Tat2 mRNA in infected cells for the mutant viruses compared with the WT viruses. The M1 mutant produced more Tat1 and Tat2 than the wild type (*P* = 0.04 and *P* = 0.05, two-tailed *t* test). The M2 mutant produced less Tat1 and Tat2 mRNA (*P* = 0.01 and *P* = 0.01). No *tat* mRNA was detected from the ERK mutant despite good amplification of the control. Graphs show means and standard errors of the means (SEM) for the results from three experiments. (C) Variations in ESE_tat_ result in different levels of Tat activity. Equal amounts of plasmids pNL4.3deltaEnv.EGFP with mutations in ESE_tat_ were transfected into TZM-bl indicator cells, which contain a Tat-driven luciferase expression cassette. Luciferase activity was measured 4 days posttransfection. Levels of signal above that seen with mock transfection were considered representative of specific Tat activity. For each experiment, the level of Tat activity was normalized to that of the WT. The graph shows means and SEM of results from five independent experiments. There was no significant difference in the levels of Tat activity between the WT and strain M1 (WT versus M1; *P* = 0.0990, two-tailed *t* test), while M2 and ERK showed progressively lower levels of Tat activity (* [WT versus M2], *P* = 0.0180; **** [WT versus ERK], *P* < 0.0001, two-tailed *t* test).

HIV *tat* mRNA is multiply spliced and has a number of different isoforms depending on the inclusion or not of small exons (Fig. 2A). All isoforms code for proteins of the same length, translated from the same initiation codon. The functional differences between various *tat* mRNA isoforms are not known, but in the work reported by Erkelenz et al. (7), disruption of the balance of *tat* mRNA isoforms resulted in inefficient viral gene expression. The predominant isoform, Tat1, is formed from the joining of the major splice donor D1 to the A3 splice acceptor, this junction site being unique to Tat1. The joining of the D2 splice donor to the A3 splice acceptor is unique to Tat2, the next most abundant isoform. We designed primer and probe pairs to detect these unique splice junctions by real-time PCR. A control primer-probe set that amplifies all *tat* and *vpr* transcripts was included to quantitate Tat1 and Tat2 levels relative to the total number of cells infected.

Intracellular RNA was extracted from Jurkat cells infected with wild-type HIV_NL4–3_ and mutants M1, M2, and ERK; levels of Tat1 and Tat2 expression were determined by quantitative PCR (qPCR). Panel B of Fig. 2 shows the expression levels of *tat* mRNA isoforms, normalized to total *tat* and *vpr* transcript levels, relative to that in the wild type. Compared to the wild type, the M1 mutant showed increased expression of Tat1 and Tat2 mRNA (*P* = 0.04 and *P* = 0.005, two-tailed *t* test) and the M2 mutant showed reduced expression (*P* = 0.01 and *P* = 0.01). Tat1 and Tat2 were not reliably detected from the ERK mutant despite good amplification of total *tat*/*vpr* in qPCR performed using the control primer-probe set.

Tat protein is expressed only to a low level and is not readily detectable by Western blotting. To assess how mutations in ESE_tat_ affect Tat activity, we cloned mutant ESE_tat_ sequences into the viral construct pNL4.3deltaEnv.EGFP and transfected the constructs into the indicator cell line Tzm-bl (Fig. 2C), which contains an HIV Tat-dependent luciferase expression cassette. The level of luciferase expression was used to assess Tat activity. There was no significant difference between the WT strain and the M1 mutant (WT versus M1; *P* = 0.0990, two-tailed *t* test) in the levels of Tat activity, while the M2 and ERK mutant strains showed progressively lower levels of Tat activity (WT versus M2, *P* = 0.0180; WT versus ERK, *P* < 0.0001 [two-tailed *t* test]).

### The degree of reduction in viral replicative kinetics is related to the extent of ESE_tat_ disruption.

**FIG 3:**
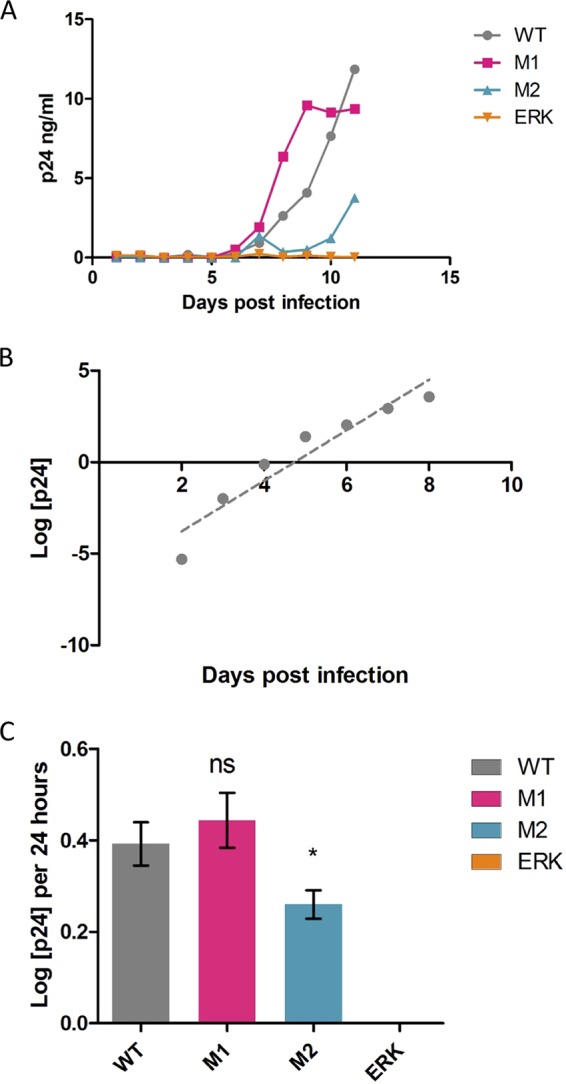
Altered growth kinetics in ESE_tat_ mutant viruses. (A) Typical data showing curves obtained from daily measurement of p24 levels in cultures infected with each virus. (B) An example of data analysis. Data determined for the logarithmic-growth phase of the WT strain reported in panel A as described above were log transformed, and a linear equation was derived. The gradient gives the log change in p24 per 24 h. (C) Reductions in virus replicative capacity were related to the degree of disruption of ESE_tat_. The replicative capacity of strain M1 was similar to that of the wild type (ns, not significant), while strain M2 showed reduced replicative capacity (0.26 log/24 h versus 0.39 log/24 h) (*, *P* = 0.04, two-tailed *t* test). Data show means and SEM of results corresponding to the log p24 per 24 h for 6 experiments. Replication of ERK was not observed in any of six experiments.

To assess replication kinetics, SupT1-CCR5 cells were infected with either full-length wild-type HIV_NL4–3_ or one of the mutants. Culture supernatants were sampled regularly for levels of viral capsid antigen p24. A typical experiment is represented in Fig. 3A; there was no detectable replication of the ERK mutant in repeated experiments.

We analyzed the rate of change of p24 levels to assess virus replicative capacity. We limited our analysis to p24 levels of 0.25ng/ml to 10 ng/ml, reflecting the log-phase period of growth. P24 measurements were log transformed, and a linear equation was derived from the data (an example is shown in Fig. 3B), with the slope giving the log change in p24 per 24 h.

There was no significant difference between the M1 mutant and wild-type virus (Fig. 3C). The M2 mutant showed slightly reduced replication kinetics (0.26 log/24 h versus 0.39log/24 h; *P* = 0.04, two-tailed *t* test). Thus, limited disruption of ESE_tat_ resulted in reduced viral replication kinetics, exemplified by M2, while severe disruption of ESE_tat_ abrogated viral replication altogether as seen in the ERK mutant.

### The extent of ESE_tat_ disruption affects the proportion of proviruses expressing viral gene products both spontaneously and upon induction.

**FIG 4:**
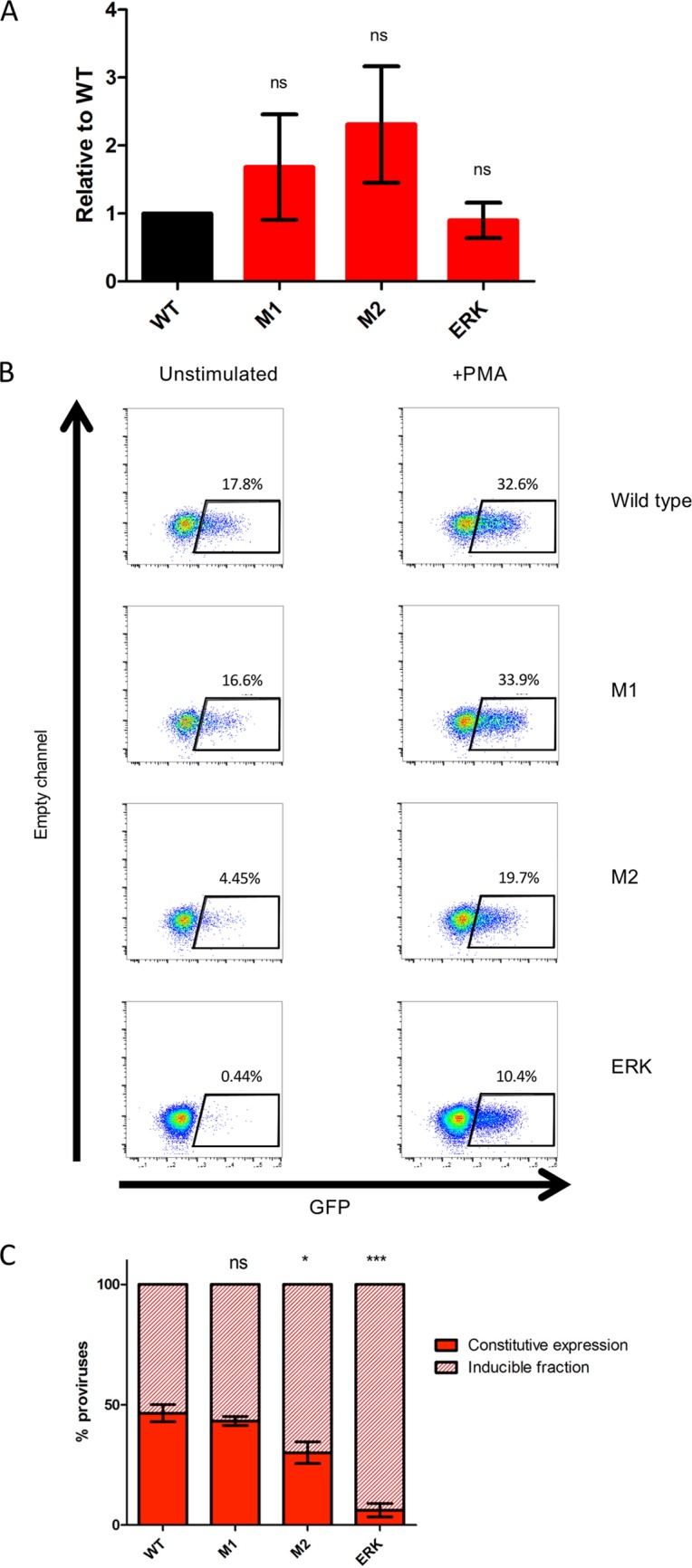
ESE_tat_ disruption increases the proportion of silent proviruses. (A) Jurkat cells were infected with GFP-expressing viruses, and DNA was harvested 4 days postinfection. Integrated proviruses were quantified using an *Alu*-PCR-based method, and the data were normalized to total LTRs to control for variations in DNA extraction and compared with WT levels. (B) Example flow plots showing unstimulated and PMA-stimulated populations of Jurkat cells infected with GFP-expressing viruses bearing the ESE_tat_ mutations. (C) The proportion of silenced proviruses increases with disruption to ESE_tat_. Solid areas represent the baseline GFP expression level as a proportion of all induced proviruses. A total of 53% of wild-type virus was silent compared to 56% for M1 (ns, *P* = 0.44 [M1 versus WT], two-tailed *t* test) and 69% for M2 (*, *P* = 0.02 [M2 versus WT]). ERK had a high (94%) proportion of silent, inducible proviruses (***, *P* = 0.00001 [ERK versus WT]). Bars show means and SEM for data from 5 experiments.

To study viral gene expression, we utilized the viral construct pNL4.3deltaEnv.EGFP, which expresses enhanced green fluorescent protein (eGFP) in place of Env, and created vectors containing ESE_tat_ mutants. The vector was pseudotyped with vesicular stomatitis virus envelope glycoprotein (VSV-G) to permit single-round infection of Jurkat cells. The levels of integrated proviruses, as detected by *Alu*-PCR (11), were similar for all four constructs (Fig. 4A).

After 72 h, the cells were stimulated with phorbol myristate acetate (PMA) in the presence of 15 nM efavirenz and 100 nM raltegravir. Those antiretrovirals prevent *de novo* infection of cells by any residual virus in the supernatant upon PMA stimulation (12), thus ensuring that inducible GFP expression arises from silent integrated constructs. Levels of viral gene expression resulting from a single infectious event can then be measured by detection of GFP expression. At 24 h after stimulation, the proportion of cells expressing GFP was quantified by flow cytometry (Fig. 4B). After PMA induction, GFP-positive populations contain both proviruses that are spontaneously expressed and those that are inducible. We can thus determine the proportions of the proviruses that are silent and of those that are inducible. For example, as indicated in the WT panel in Fig. 4B, 17.8% of the cells expressed GFP before stimulation and 32.6% poststimulation. In this example, 45% [(32.6 − 17.8)/32.6] of all PMA inducible integration events were initially silent, while 55% expressed spontaneously.

The data demonstrate a clear trend, indicating that the proportions of silent (but inducible) proviruses and of active proviruses are related to the degree of disruption of ESE_tat_. A total of 53% of the wild-type virus was silent (Fig. 4C) compared to 56% for M1 (*P* = 0.44, M1 versus WT, two-tailed *t* test) and 69% for M2 (*P* = 0.02, M2 versus WT). To our surprise, ERK could be induced to express upon exposure to high doses of PMA but demonstrated the presence of a high (94%) proportion of silent but inducible proviruses (*P* = 0.00001, ERK versus WT).

### Inducibility is not a binary phenotype and can be related to the degree of disruption of the provirus.

**FIG 5:**
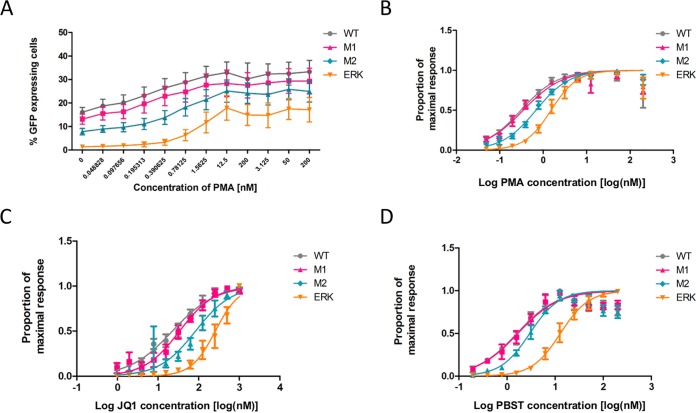
ESE_tat_ mutant viruses exhibit a higher threshold for reactivation from latency. (A) Jurkat cells infected with GFP-expressing viruses were stimulated with a 2-fold dilution series of PMA. The percentage of GFP-positive cells was determined by flow cytometry. The graph demonstrates the percentage of GFP-expressing cells at each concentration of PMA for each of the four viruses. Each data point shows means and SEM for data from 5 experiments. (B) Mutant viruses exhibit a higher threshold for reactivation with PMA. Dose-response curves were fitted to the data using the maximum and minimum percentages of GFP to derive the EC_50_, i.e., the dose required for 50% response. EC_50_ values were higher in M2 and ERK viruses. (C) Dose-response curves for the latency-reversing agent JQ1 showing an increased threshold of activation for M2 and ERK (*n* = 3). (D) Dose-response curves for panobinostat (PBST) showing an increased threshold of activation for the ERK virus (*n* = 3).

We hypothesized that the ability of a silent provirus to be induced to express viral gene products does not represent a binary phenotype. In other words, the level of stimulus required to induce expression ranges along a spectrum and the degree of inducibility can be assessed by analysis of the threshold required to reactivate the silent provirus. The induction threshold would be reflected in the concentration of latency reversal agents required to achieve gene expression from a silent provirus. The ESE_tat_ model was used to evaluate this. Pseudotyped, GFP-expressing HIV vector with wild-type and mutated ESE_tat_ sequences was used to infect Jurkat cells. After 72 h, the medium was replaced with one containing 100 nM raltegravir and 15 nM efavirenz as well as PMA in serial 2-fold dilutions at concentrations between 0.048 nM and 200 nM. An infected but unstimulated well was included in each experiment to represent baseline GFP expression. At 24 h after stimulation, GFP expression was determined by flow cytometry. Panel A of Fig. 5 shows the mean percentages of GFP-expressing cells observed at each concentration of PMA for 5 experiments.

To compare the thresholds of reactivation of different viruses, dose-response curves were fitted to the data. The data were scaled such that the level of baseline expression in the absence of PMA was set to 0 and the maximum observed level of GFP expression was set to 1. Other values were scaled accordingly (Fig. 5B). The analysis was used to compute the effective concentration of PMA required to achieve 50% maximal reactivation (EC_50_). Table 1 shows the EC_50_ for each mutant derived using this method. Confidence intervals were used to calculate a *T* statistic and therefore a *P* value for comparisons to the wild type. There was no statistically significant difference between the WT and M1 viruses. However, the EC_50_ seen with the M2 virus was significantly higher (0.6797 nM versus 0.3074 nM, *P* = 0.025, M2 versus WT) and that seen with the ERK mutant was higher still (1.517 nM, *P* = 0.0011, ERK versus WT; *P* = 0.004, ERK versus M2).

**TABLE 1:** PMA EC_50_ values for each virus^*a*^

Strain	EC_50_ (nM)	95% confidence interval	*P* value
WT	0.3074	0.2263 to 0.4174	
M1	0.3472	0.2658 to 0.4534	0.63
M2	0.6797	0.5824 to 0.7931	0.025
ERK	1.517	1.226 to 1.877	0.0011

a The PMA EC_50_ values for each virus were determined as described in the Fig. 5 legend. Confidence intervals were used to derive a *T* statistic and a *P* value for the comparison with the wild type. There were no significant differences in the EC_50_ values for the M1 mutant, but the EC_50_ values were higher for both the M2 and ERK mutants.

The effect on reactivation by other latency-reversing agents, including the bromodomain inhibitor JQ1 (Fig. 5C) and a clinically relevant histone deacetylase (HDAC) inhibitor, panobinostat (13) (Fig. 5D), was also studied. Similarly to the dose response observed with PMA, there were no significant differences in EC_50_ levels for the WT and M1 strains for both JQ1 and panobinostat. The threshold to achieve reactivation shown by the M2 and ERK viruses with JQ1 was higher than that seen with the wild type (Table 2; 83.7 nM versus 22.58 nM [*P* = 0.0258] and 256.8 nM versus 22.58 nM [*P* = 0.0035]). There was a significant increase in the threshold for reactivation seen with panobinostat for the ERK mutant (Table 3; 12.86 nM versus 1.6 nM, 0.0084). There was also a trend toward an increase seen with the M2 mutant, but the data were not statistically significant. Thus, with a number of latency reversal agents, the concentration required to achieve an equivalent level of response increased with more-extensive disruption of ESE_tat_, reflecting a higher threshold of reactivation and a lower level of inducibility.

**TABLE 2:** JQ1 EC_50_ values for each virus^*a*^

Strain	EC_50_ (nM)	95% confidence interval	*P* value
WT	22.58	18.09 to 28.17	
M1	33.88	26.43 to 43.43	0.11
M2	83.7	57.37 to 122.1	0.0258
ERK	256.8	211.3 to 312.1	0.0035

a JQ1 EC_50_ values for each virus were determined as described in Fig. 5. There were no significant differences in the EC_50_ values for the M1 mutant, but the EC_50_ values were higher for both the M2 and ERK mutants.

**TABLE 3:** Panobinostat EC_50_ values for each virus^*a*^

Strain	EC_50_ (nM)	95% confidence interval	*P* value
WT	1.603	1.118 to 2.299	
M1	1.68	1.204 to 2.344	0.864
M2	2.972	2.226 to 3.969	0.11
ERK	12.86	11.01 to 15.03	0.0084

a Panobinostat EC_50_ levels for each virus were determined as described in Fig. 5. There were no significant differences in the EC_50_ values for the M1 mutant. There was a small but nonsignificant difference in the EC_50_ level for the M2 mutant. The ERK mutant required a significantly higher concentration of panobinostat.

## DISCUSSION

We found that viral replicative capacity shows a strong negative correlation with progressively increased disruption of the viral splicing regulatory element ESE_tat_. A more functionally defective form of ESE_tat_ is associated with a higher proportion of integrated constructs being silenced; once silenced, the threshold for reactivation by exogenous stimuli is also higher. Thus, inducibility is not a binary phenotype—a more extensively disrupted provirus is less inducible, while a virus with a level of disruption closer to that of the wild type is more inducible. Of note, our data show that the effect seen with the mutants was not due to differences in the levels of provirus. Importantly, as we made this observation on the basis of the relative proportions of cells expressing the viral vectors with or without induction, our conclusion is not affected by any effect ESE_tat_ may have on other parts of the virus life cycle.

While host factors affecting HIV latency have been studied extensively, the contribution of viral factors is less well explored. In the present model, subtle disruption of the Tat/TAR axis results in a viral phenotype that is reminiscent of latency. This is consistent with previously described cell line models of HIV latency, including ACH-2, in which a mutation in the TAR loop abrogates binding of Tat and prevents transactivation (14, 15), and HIV mutant Tat H13L, which is less able to bind positive transcription elongation factor b (PTEF-b), essential for Tat activity (16). However, it is unclear whether these examples of functional impairment generated *in vitro* reflect the biology of HIV latency *in vivo*. Here, we studied naturally occurring polymorphisms in a recently identified viral mRNA splicing regulatory element, ESE_tat_. We observed that the ability of the virus to reactivate is related to the degree of disruption of viral mRNA splicing regulation. We have thus provided an example of an inefficient posttranscriptional process resulting in a latency-like phenotype; it is likely that modulation of mRNA splicing represents only one mechanism by which this can occur.

We have not explored the mechanisms underpinning the link between ESE_tat_ disruption and inefficient viral gene expression. Erkelenz et al. (7), whose publication originally identified and described the *tat* exonic splicing enhancer, demonstrated that disruption of ESE_tat_ resulted in a marked reduction in the level of *tat* mRNA production with corresponding levels of preservation of singly and unspliced transcripts. They also demonstrated that their disruption abrogated the binding of splicing factors serine- and arginine-rich splicing factor 2 (SRSF2) and SRSF6. Here, we sought not to explore the efficiency of translation of different isoforms of tat mRNA but to use the polymorphisms as a model to demonstrate that apparently minor mutations in the viral expression machinery can affect how readily a latent virus can be induced to express. Our data show that a more easily inducible virus also has a higher replicative capacity. In contrast, the associations among viral defectiveness, replication capacity, and inducibility may not hold true if the defect affects aspects of viral life cycle other than gene expression.

How can our findings be applied to HIV latency *in vivo*? The model described here is based on viruses generated *in vitro*. *In vivo*, latent HIV is genetically heterogeneous (17). The basis of this heterogeneity is error-prone reverse transcription, possibly with additional mutations introduced by the apolipoprotein B mRNA editing enzyme catalytic polypeptide-like (APOBEC) system of the host cell (18). These processes happen before incoming HIV is archived as a latent provirus. Hence, *in vivo*, latent proviruses are likely to be genetically diverse; some of them inevitably have mutations affecting viral gene expression through sheer chance. As demonstrated with the current model, and consistent with previous reports (19), these disruptions do not necessarily have to directly involve the viral promoter regulating viral transcription but can involve any component of the complex apparatus involved in viral gene expression, including transcription initiation, production and splicing of early transcripts, and Tat transactivation. Thus, it is more than probable that nondefective latent proviruses would have a spectrum of inducibility *in vivo*, reflecting the degree of disruption accrued in the virus gene expression apparatus. These disruptions might affect any stage of the postintegration life cycle, from altered binding of transcription factors at the promoter to differential translation of mRNA isoforms. Indeed, others have constructed isogenic mutants of HIV_LAI_ containing the promoters of different HIV subtypes and found that the promoter of subtype AE had a higher level of basal activity but was less responsive to tumor necrosis factor alpha (TNF-α) than was the case with subtype B viruses (20). The promoters of HIV are heterogeneous, and the numbers of NFκB binding sites differ for different subtypes. In addition, polymorphism of an AP-1 binding site has been found to affect the silencing and reactivation of HIV (21). Emery et al. (22) studied HIV RNA splicing with a panel of subtype B transmitter/founder viruses and a subtype C virus and noted considerable variability in the use of the A3/tat splice acceptor site. This could also potentially have an impact on the silencing behavior and inducibility of the virus. Latency and inducibility thus represent complex phenotypes that reflect an aggregate of viral and host factors, and our model is a demonstration of how perturbing of just one facet of the transcriptional machinery can lead to higher rates of silencing and serves as an exemplar of a viral factor enhancing silencing. Future studies may identify a range of other mechanisms that produce similar phenotypes.

The “shock and kill” (23) strategy aims to achieve HIV cure through therapeutic reactivation of latent HIV to provide targets for immune clearance (24, 25). If viral reactivation represents not a binary but a continuous variable, then such reactivation may become increasingly difficult with successive rounds of stimulation, as latent proviruses with lower thresholds of reactivation respond to earlier rounds of activation, leaving proviruses with higher thresholds in the reservoir.

Here we have demonstrated that disrupting HIV gene expression by altering *tat* mRNA splicing can cause an inducible and variable proviral silencing phenotype comparable to and superficially indistinguishable from viral latency. We found that inducibility is not a binary phenotype and that the threshold of reactivation of silent proviruses varies with the extent of disruption of the virus. This has potentially significant implications for the shock and kill approach to HIV cure.

## MATERIALS AND METHODS

### Plasmids.

Mutations in ESE_tat_ were introduced by site-directed mutagenesis. A 1.8-kb fragment between the EcoR1 and Nhe1 restriction sites of pNL4.3 was cloned into the corresponding sites in plasmid pBR322. Mutagenesis was carried out using a QuikChange II kit (Agilent) and the manufacturer’s protocol. Mutagenesis was confirmed by Sanger sequencing. Mutated fragments were cloned back into pNL4.3 or pNL4.3.deltaENV.EGFP (both from the NIH AIDS Reagent Program). pCMV-VSVG (Addgene) was used to supply vesicular stomatitis virus envelope glycoprotein for pseudotyping the GFP-expressing virus.

### Cell culture.

SupT1-CCR5 cells were a kind gift from James Hoxie of the University of Pennsylvania. Jurkat cells and SupT1-CCR5 cells were maintained in RPMI medium supplemented with 10% fetal calf serum and 100 units/ml penicillin and streptomycin (all from Gibco). HEK 293T cells and Tzm-bl cells were both maintained in Dulbecco’s modified Eagle’s medium (DMEM) (Gibco) supplemented as described above. Transfection of 293T cells was carried out using the calcium phosphate method. Transfection of Tzm-bl cells was carried out with Lipofectamine 2000 (Thermo Fisher).

### Luciferase assay.

Tzm-bl cells were harvested 4 days after transfection. Culture supernatant was removed, and the cells were washed with phosphate-buffered saline (PBS) before lysis with CCLR (Promega). Luciferase activity was detected by GloMax analysis using a standard luciferase detection kit (Promega). Transfection experiments were conducted in duplicate, and for each transfected well, luciferase detection was also conducted in duplicate. Specific signal data were calculated by subtracting the readout from mock-transfected wells from the readout from wells transfected with viral constructs, and data were normalized to the WT virus.

### Virus stocks.

Virus stocks were prepared from the supernatants of transfected HEK 239T cells. The virus was concentrated by ultracentrifugation, and titers were determined using p24 enzyme-linked immunosorbent assay (ELISA). Stocks were frozen at −70°C after the titers were determined.

### RNA extraction and qPCR.

RNA was extracted from cells 48 h after infection using an RNeasy Plus minikit (Qiagen) according to the manufacturer's protocol. cDNA was prepared using a High-Capacity cDNA reverse transcription kit (Thermo Fisher) and cleaned up using a DNA purification kit (Qiagen). Water-only and no-RT controls were included in the experiments as negative controls. Quantitative PCR was carried out with qPCR Mastermix (Applied Biosystems) following the manufacturer’s instructions on a StepOnePlus real-time PCR system (Applied Biosystems) using the following cycling parameters: 1 cycle at 95°C for 8 min and 45 cycles at 95°C for 10 s followed by 60°C for 1 min. The primers and probes (Sigma) used were as follows: Tat1 forward, 5′-AGA TCT CTC GAC GCA GGA CT-3′; Tat1 reverse, 5′-GGC TGA CTT CCT GGA TGC TT-3′; D1A3 probe (tat1), 5′-6-carboxyfluorescein (FAM)-TCG ACA CCC AAT TCA GTC GC-6-carboxytetramethylrhodamine (TAMRA)-3′; Tat2 forward, 5′-GGA CAG CAG AGA TCC AGT TTG-3′; Tat2 reverse, 5′-GAT GCT TCC AGG GCT CTA GTC-3′; D2A3 probe (tat2), 5′-FAM-GTC GAC ACC CAA TTC TTT CCA G-TAMRA-3′; All-tat/vpr forward, 5′-TCC TAT GGC AGG AAG AAG CG-3′; All-tat/vpr reverse, 5′-AGC TTG ATG AGT CTG ACT GT-3′; All-tat/vpr probe, 5′-FAM-TCT GAT GAG CTC TTC GTC GCT GTC TC-TAMRA-3′.

### DNA extraction and qPCR.

DNA was extracted from cells 4 days after transduction using a DNeasy blood and tissue minikit (Qiagen). DNA extract was diluted 1:10 and subjected to *Alu*-PCR as previously described (11). Briefly, first-round PCR was conducted with a lambda-long terminal repeat (lamba-LTR) primer with or without *Alu* primer. The reaction parameters for first-round PCR were as follows: 1 cycle at 95°C for 8 min followed 12 cycles at 95°C for 30 s, 60°C for 10 s, and 72°C for 3 min followed by 1 cycle at 72°C for 7 min. The levels of integrated constructs are expected to increase exponentially with this round of PCR, while the levels of unintegrated construct are expected to increase linearly. PCR products were cleaned up using a PCR purification kit (Qiagen) and subjected to specific lambda-LTR quantitative PCR, with cycling parameters as described in the previous section. The sequences for primers and probes were identical to previously published sequences (11), with the only modification being the use of FAM and TAMRA as fluorophores. To control for variations in DNA extraction efficiency, the readout was normalized to that of a total LTR PCR, using the following primers and probe (Sigma): for primer 457F, 5′-TGG GAG CTC TCT GGC TAA CT-3′; for primer 615R, 5′-GGC GCC ACT GCT AGA GAT TT-3′; for probe 520R, 5′-FAM-CAC TCA AGG CAA GCT TTA TTG AGG C-TAM-3′.

### Replication kinetics analysis.

A total of 5 to 8 million SupT1-CCR5 cells were infected with 15 ng p24 per virus by spinoculation. Infected cells were washed three times with PBS to remove the inoculum before resuspension in culture media. Supernatant was sampled every 24 h after infection and stored; at the end of the experiment, supernatant p24 levels were determined by ELISA.

### Reactivation assays.

A 50-ng volume of p24 of pseudotyped virus was used to infect 5 × 10^5^ Jurkat cells. After 72 h, the cells were seeded into 12 wells of a 96-well plate. The medium was removed from the cells and replaced with medium containing 100 nM raltegravir and 15 nM efavirenz together with any latency reversal agents to be tested in serial 2-fold dilutions. An infected but unstimulated well was included in each experiment as a measure of baseline GFP expression. PMA (Sigma), panobinostat (Sigma), and JQ1 (Cayman) were used at the indicated concentrations. Efavirenz and raltegravir were obtained from the NIH AIDS reagents program. Flow cytometry data were collected using an Attune NXT flow cytometer (Thermo Fisher), and flow cytometry plots were analyzed using FlowJo 10.
